# Clinical features of acute generalized exanthematous pustulosis caused by hydroxychloroquine in rheumatology patients and exploration of *CARD14* gene mutations

**DOI:** 10.3389/fmed.2023.1161837

**Published:** 2023-04-06

**Authors:** Feng Luo, Xue-mei Yuan, Hong Xiong, Yu-zheng Yang, Chang-ming Chen, Wu-kai Ma, Xue-ming Yao

**Affiliations:** ^1^Graduate School, Guizhou University of Traditional Chinese Medicine, Guiyang, China; ^2^Department of Rheumatology and Immunology, Second Affiliated Hospital of Guizhou University of Chinese Medicine, Guiyang, China

**Keywords:** acute generalized exanthematous pustulosis, hydroxychloroquine, *CARD14*, gene mutation, gene sequencing

## Abstract

**Introduction:**

Acute generalized exanthematous pustulosis (AGEP) is a rare condition characterized by superficial pustules following drug ingestion or infection. Currently, there is no clear link between rheumatism and AGEP. It has been described that hydroxychloroquine (HCQ) is a rare cause of acute generalized epidermal necrolysis (AGEP). Presently, there are limited studies on HCQ-induced AGEP. We aimed to explore the clinical features and associated gene expression of AGEP induced after HCQ treatment exposure in rheumatology patients.

**Methods:**

We assessed patients with HCQ-induced AGEP diagnosed at the Second Affiliated Hospital of Guizhou University of Chinese Medicine between January 1, 2017, and May 1, 2022. We also reviewed similar cases reported in specific databases.

**Results:**

The study included five females (mean age, 40.2 years), and the mean time from initiation of HCQ treatment to symptom onset was 12.2 d. All patients received steroids and allergy medications after HCQ discontinuation, and the rash completely resolved within an average of 25.2 d. We performed whole exome sequencing and Sanger validation in our patient sample. *CARD14* gene mutations were detected in three patients. Additionally, seven mutation sites were detected.

**Discussion:**

HCQ-induced AGEP may have a longer latency period and regression time than AGEP induced by other drugs. Our patients all experienced *CARD14* gene mutations. AGEP often resolves with topical therapy and drug discontinuation, although some cases require systemic steroid therapy. In the future, patients with rheumatism should pay attention to the effectiveness of HCQ during treatment and be aware of the associated skin toxicity.

## 1. Introduction

A rheumatic disease is an inflammatory disorder that affects the skin, mucous membranes, bone, joints, and surrounding soft tissues including muscles, synovium, bursa, and tendons (1). The most common symptom of acute generalized exanthematous pustulosis is the presence of numerous non-follicular, sterile pustules on top of an edematous background (2). It is usually accompanied by fever and peripheral blood leukocytosis (3). About 90% of AGEP cases are caused by drugs such as antibiotics, antifungals, diltiazem, and antimalarial agents (4, 5). Approximately 20% of patients with AGEP develop systemic involvement; mostly impaired liver, kidney, lung function and, in severe cases, multiple organ failure, diffuse intravascular coagulation, and death (6, 7). Hydroxychloroquine (HCQ) sulfate regulates immune function and can be used clinically as initial treatment for malaria, rheumatoid arthritis (RA), and systemic lupus erythematosus (SLE) (8, 9). With the widespread use of HCQ, an increasing number of adverse events are being reported in clinical practice, among which AGEP is rare. Owing to its low incidence, we have limited information on AGEP. Previous research has demonstrated that some patients with AGEP carry mutations in the *IL36RN* and *CARD14* genes, suggesting that AGEP may have a genetic basis (10). In addition, there is evidence that AGEP is linked to mutations in the *CARD14* gene, and the predicted p. (Arg430Trp) variant of heterozygous c.1288C c.1288C > T transition (11). We conducted a literature search and found only a few reported cases of Chinese clusters (12, 13). Therefore, the study aimed to describe the clinical and genetic characteristics of Chinese patients with HCQ-induced AGEP.

## 2. Materials and methods

We observed five patients of HCQ-induced AGEP at the Second Affiliated Hospital of Guizhou University of Chinese Medicine between January 1, 2017, and May 1, 2022. The patients were diagnosed with HCQ-induced AGEP based on EuroSCAR scores validated by (5); five patients scored 5–12 points, indicating a definite or probable diagnosis of AGEP (5). We collected the following information from the medical records of these patients: demographic characteristics, clinical features, onset date, time of symptom resolution, and laboratory test results. In addition, we used next-generation whole exome sequencing to evaluate the mutated gene, and performed Sanger verification on the mutant gene; *IL36RN* and *CARD14* mutation sites were reported. Our gene sequencing experiments were all entrusted to the Beijing Luhe Huada Gene Technology Co., Ltd., Wuhan, China, and the specific process can be found on the company’s website.^1^ Furthermore, we reviewed similar cases reported in the Wanfang Data, PubMed, Embase, China National Knowledge Infrastructure (CNKI), Web of Science, and other databases from their inception to May 2022. Search terms included “acute generalized exanthematous pustulosis,” “AGEP,” “HCQ,” “hydroxychloroquine,” and “side effects.” The present study was approved by the Institutional Review Committee of the Second Affiliated Hospital of Guizhou University of Chinese Medicine and conducted in accordance with the Declaration of Helsinki. Throughout the study, all participants provided their informed consent.

## 3. Results

The average age at diagnosis was 40.2 years (range, 28–63 years) for all five participants. Patient history included two cases of RA, one case of RA with Sjogren’s syndrome, and two cases of SLE. None of the five patients had any skin reactions or a personal or family history of psoriasis. The details of each case are presented in Table 1.

**Table 1:** Clinical characteristics, laboratory abnormalities, and allergological findings.

Age/sex	Primary disease	Delay to onset of symptoms	Co-medication	Fever	Cutaneous involvement	Neutrophilia	Eosinophilia	Treatment	Resolution	Specific mutation
32/F	SLE	15 d	Low dose steroid/calcium carbonate and vitamin D3/pantoprazole sodium enteric-coated (long-term use)	Yes	Initially affecting the limbs, an itching pustular rash with an erythematous base that spread to other parts	No	No	Systemic steroids/antihistamine/mycophenolate mofetil/thalidomide	41 d	No
33/F	RA	7 d	Methotrexate/folic acid (long-term use)	No	Pustules with an erythematous base that started on the scalp and spread throughout the body	No	No	Topical steroid/antihistamine	15 d	No
63/F	SLE	9 d	Low dose steroid/calcium carbonate/pantoprazole sodium enteric-coated	Yes	Itchy pustular rash with an erythematous base, beginning on the face and spreading to the body	Yes	No	Systemic steroids/antihistamine	19 d	NM_001366385.1(CARD14):c.1641G > C (p.Arg547Ser); NM_001366385.1(CARD14):c.2458C > T (p.Arg820Trp); NM_001366385.1(CARD14):c.2422A > G (p.Thr808Ala); NM_001366385.1(CARD14):c.1323C > T (p.Asp441Asp).
28/F	RA	20 d	Leflunomide (long-term use)	Yes	Itching pustular rash with an erythematous base that started on the hand and spread to other parts	Yes	No	Systemic steroids/antihistamine	24 d	NM_001366385.1(CARD14):c.2458C > T (p.Arg820Trp); NM_001366385.1(CARD14):c.2422A > G (p.Thr808Ala).
45/F	RA/pSS	25 d	Low dose steroid/pantoprazole sodium enteric-coated/iguratimod	Yes	Itchy pustular rash with an erythematous base, beginning on the face and spreading to the body	Yes	No	Systemic steroids/antihistamine	36 d	NM_001366385.1(CARD14):c.1753G > A (p.Val585Ile); NM_001366385.1(CARD14):c.633G > A(p.Glu211Glu); NM_001366385.1(CARD14):c.2481C > T (p.Pro827Pro)

F, female; pSS, primary dryness syndrome; RA, rheumatoid arthritis; SLE, systemic lupus erythematosus.

**Figure 1:**
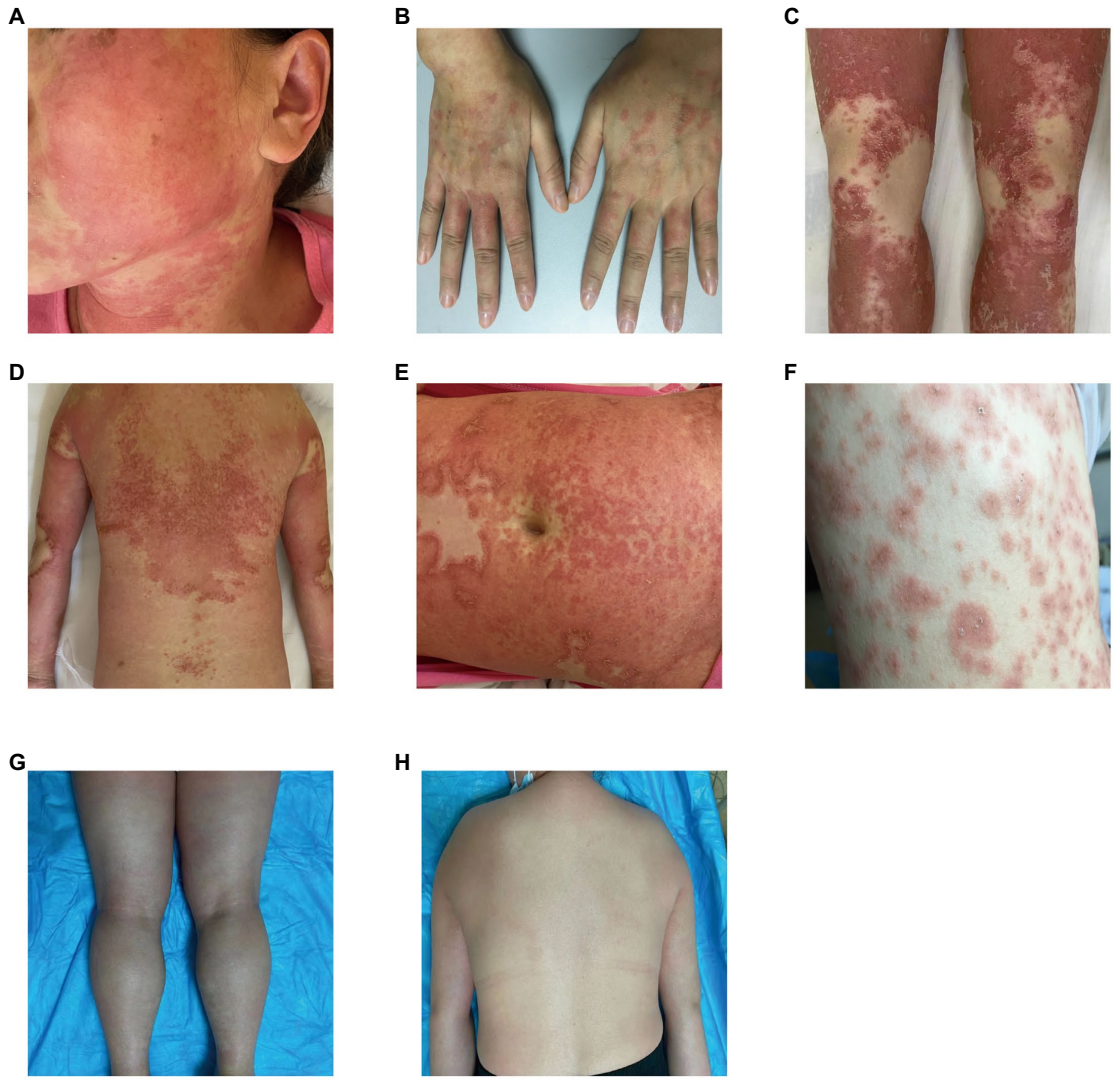
Rash before and after treatment. **(A)** Rash on the face; **(B)** rash on the hands; **(C)** rash on the legs; **(D)** rash on the back; **(E)** rash on the abdomen; **(F)** rash on the chest; **(G)** improvement in the rash on the legs; and **(H)** improvement in the rash on the back.

Our five cases were accustomed to hospital treatment because of a poor response to the long-term use of disease-modifying antirheumatic drugs (DMARDs), such as methotrexate for rheumatism, and skin allergic reactions caused by adjusting to the use of HCQ. The dosage of HCQ among the five patients was the same: two tablets, administered twice daily at the same time. The average delay in the appearance of a rash in all five cases was 12.2 d after admission (range: 7–25 d). In all patients, the skin eruptions were non-follicular and pinhead-sized, resulting from sudden, acute onset. The rash was initially seen on the cheeks and face in two patients, on a limb joint in one patient, on the scalp in one patient, and on the hands in one patient. In all cases, a rapid spread of the rash throughout the body was observed, with facial involvement (Figures 1A–F). After the rash appeared, all patients immediately stopped HCQ treatment. No patients developed mucosal involvement or lymphatic system disease. Over the course of symptom duration, four patients developed fever and all patients had pruritis.

Laboratory results showed that the percentage of neutrophils increased to 81.3% (normal, 40–75%) in three patients, and the percentage of eosinophils decreased to 0.3% (normal, 0.4–8.0%) in two patients. C-reactive protein levels increased to 11.21 mg/L (the norm, 0–10 mg/L) in one patient, and the erythrocyte sedimentation rate increased to 59 mm/L (normal, 0–20 mm/L) in one patient. Several routine laboratory tests were also performed, including renal and hepatic function and serologic tests to determine influenza, hepatitis B, enteroviruses, and mycoplasmas, which were unremarkable. The bacteriological and mycological findings of pustules were negative.

**Figure 2:**
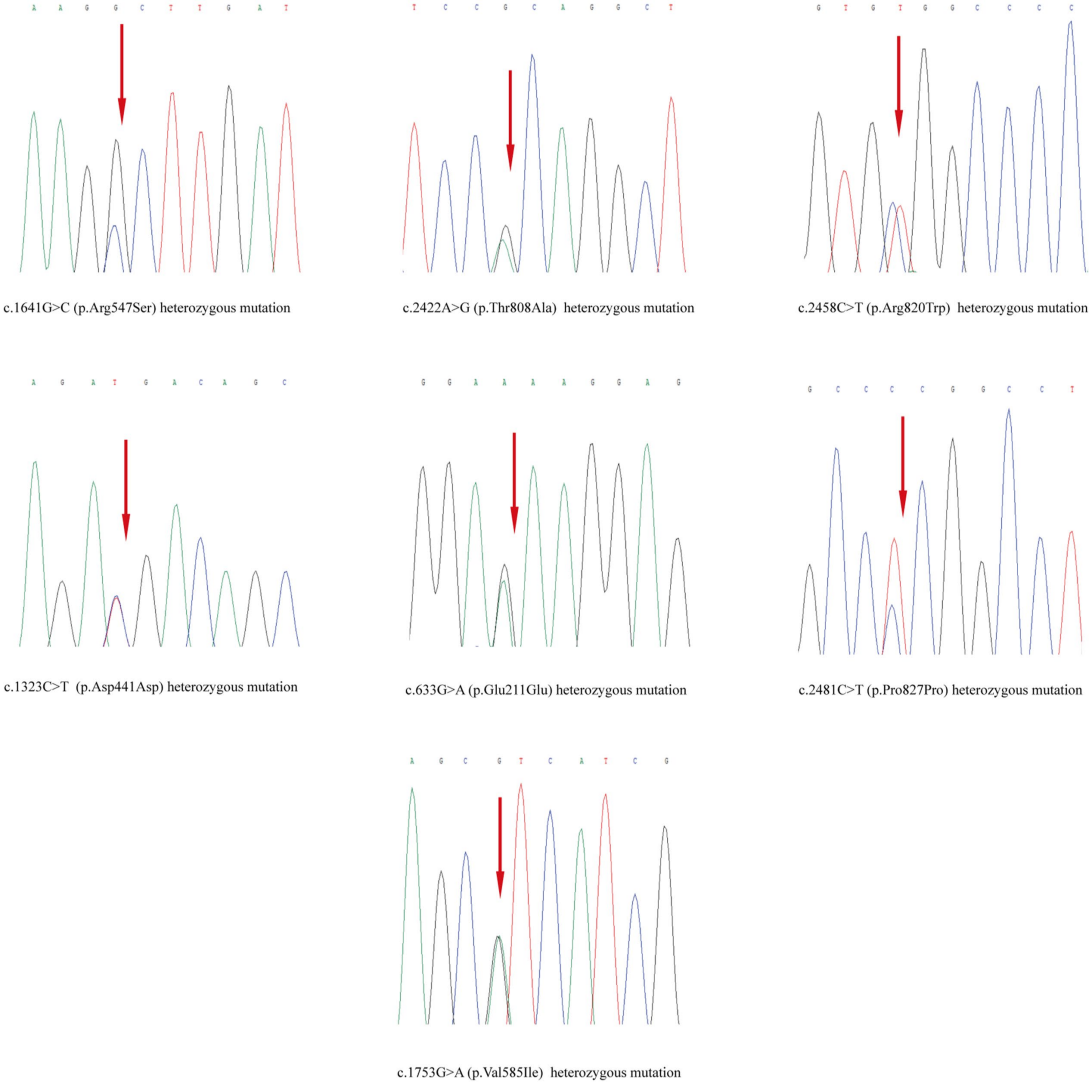
Histopathological examination of a skin biopsy from the left chest showed mild vacuolar degeneration of epidermal basal cells, varying numbers of neutrophil infiltrates in the superficial-deep dermis (mainly in the superficial dermis), and epidermal cavernous pustule formation (hematoxylin and eosin staining; original magnification, ×400).

**Figure 3:**
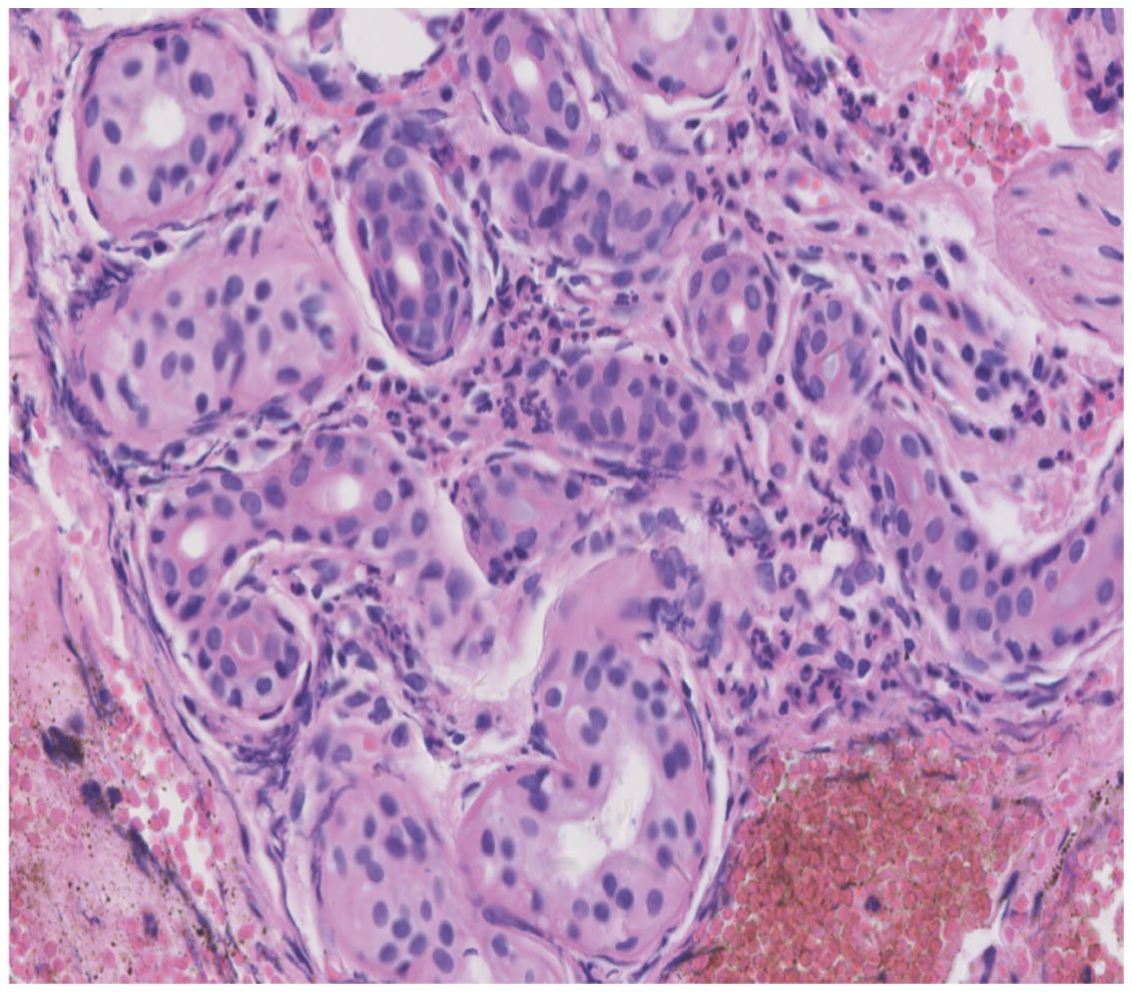
Mutation sites detected in the samples.

Skin tissue biopsy showed histopathological changes in the skin, where the epidermis showed mild vacuolar degeneration and varying numbers of neutrophil infiltrates just beneath the surface of the deep dermis—primarily the superficial dermis—as well as cavernous pustules under the epidermis; an example of these changes is presented in Figure 2. We performed whole exome sequencing and Sanger validation on all five patients and identified *CARD14* gene mutations in three patients. Seven mutation sites were detected: c.1641G > C (p.Arg547Ser) heterozygous mutation, c.2422A > G (p.Thr808Ala) heterozygous/homozygous mutation, c.2458C > T (p.Arg820Trp) heterozygous mutation, c.1323C > T (p.Asp441Asp) heterozygous mutation, c.633G > A (p.Glu211Glu) heterozygous mutation, c.1753G > A (p.Val585Ile) heterozygous mutation, and c.2481C > T (p.Pro827Pro) heterozygous mutation (Figure 3). No mutations were identified in the *IL-36RN* gene.

One patient was treated with topical steroids plus antihistamines, three patients received systemic steroids plus antihistamines, and one patient was treated with steroids plus antihistamines, mycophenolate mofetil, and thalidomide. All patients achieved good results after treatment, the rash completely resolved within 15–45 d after HCQ discontinuation, and diffuse superficial desquamation was observed during treatment. All patients were followed-up for 6 months to 1 year, without rash recurrence (Figures 1G,H).

From our literature search strategy, 221 relevant articles were retrieved. After screening, 33 valid articles were obtained, including 46 similar cases. Table 2 shows the clinical features of these cases. The cases we reported were similar to those reported in the literature. These similarities concerned the greater proportion of females, average age of onset and mean delay to diagnosis, treatment with topical or systemic steroids, and complete rash resolution within 7–119 d.

**Table 2:** Previous reports on cases of acute generalized exanthematous pustulosis induced by HCQ.

Study	Number of cases	Age/Sex	Primary disease	Delay to onset of symptoms	Treatment	Resolution after HCQ-withdrawal
Di Lernia et al. (14)	1	63/F	RA	30 d	Systemic steroids/cyclosporine	55 d
Liccioli et al. (15)	1	9/F	Juvenile Sjogren’s syndrome	30 d	No treatment	7 d
Di Maso et al. (16)	1	34/F	Undifferentiated connective tissue disease	2 d	Systemic steroids/CPFA	Not reported
Soria et al. (17)	6	48/23/45/F 9/52/M	Erythematous facialdermatitis/Photosensitivity/SLE/RA/Cutaneous lupus erythematosus	3–18 d	Not reported	7–18 d
Pearson et al. (18)	1	50/F	RA	14 d	Topical steroid	81 d
İslamoğlu et al. (19)	1	64/F	pSS	20 d	Systemic steroids/cyclosporine	37 d
Matsuda-Hirose et al. (20)	1	31/F	SLE	15 d	Systemic steroids	119 d
Bailey et al. (21)	1	48/F	SLE	14 d	Systemic steroids	18 d
Assier-Bonnet et al. (22)	1	36/F	Seronegative polyarthritis	12 d	No treatment	13 d
Evans et al. (23)	1	28/F	SLE	15 d	No treatment	21 d
Martins et al. (24)	1	51/F	RA	14 d	Systemic steroids	21 d
Paradisi et al. (25)	3	36/F 70/79 M	RA and pSS/RA/polymyalgia rheumatica	21/20/20 d	Systemic steroids	8 d/12 d/15 d
Avram et al. (26)	1	79/F	RA	14 d	No treatment	14 d
Park et al. (27)	1	38/F	Dermatomyositis	20 d	Systemic steroids	Not reported
Charfi et al. (28)	1	33/F	SLE	17 d	No treatment	7 d
Zhang et al. (29)	1	60/F	pSS	25 d	Systemic steroids	14 d
Yalcin et al. (30)	1	67/F	Seronegative polyarthritis	15 d	Systemic steroids/cyclosporine	21 d
Castner et al. (31)	1	56/F	pSS	21 d	Systemic steroids/cyclosporine	17 d
Duman et al. (32)	1	42/F	RA	20 d	Systemic steroids	35 d
Mohaghegh et al. (33)	1	44/F	Distal joint pain	5 d	Topical steroids	68 d
Chaabouni et al. (34)	7	47 (range: 35–64)/7F	pSS (three cases)/lichen planus (one case)/recurrent aphthous stomatitis (one case)/systemic scleroderma (one case)/Melkersson–Rosenthal syndrome (one case)	40 d (range: 15–122 d)	Topical steroids (seven cases)	39 d (range: 15–91 d)
Mofarrah et al. (35)	1	49F	RA	17 d	Topical steroids	7 d
Munshi et al. (36)	1	76 M	Calcium pyrophosphate dihydrate crystal deposition disease	18 d	Ixekizumab	11 d
Otake-Irie et al. (37)	1	61 M	SLE	7 d	Systemic steroids	70 d
Enos et al. (38)	1	29F	Stevens-Johnson syndrome	4 d	Systemic steroids	38 d
Mercogliano et al. (39)	1	71/F	RA	14 d	No treatment	13 d
Robustelli et al. (40)	1	70/F	SARS-CoV-2 pneumonia	10 d	Topical steroids	30 d
Litaiem et al. (41)	1	39/F	SARS-CoV-2 pneumonia	18 d	Died of pulmonary embolism	-
Sánchez-Velázquez et al. (42)	1	31/F	COVID-19	9 d	Cyclosporine	40 d
Lateef et al. (43)	1	67/F	SLE	21 d	Systemic steroids	14 d
Coleman et al. (44)	1	68/F	Stevens-Johnson syndrome	20 d	Systemic steroids/intravenous immunoglobulin	10 d
Liu et al. (13)	1	69/F	pSS/hypertension/pulmonary fibrosis	20 d	Systemic steroids	16 d
Tian et al. (12)	1	35/F	Undifferentiated connective tissue diseases	10 d	Systemic steroids/cyclosporine	29 d

COVID-19, coronavirus disease 2019; CPFA, coupled plasma filtration adsorption; F, female; HCQ, hydroxychloroquine; M, male; pSS, primary dryness syndrome; RA, rheumatoid arthritis; SLE, systemic lupus erythematosus.

## 4. Discussion

AGEP is an acute-onset non-follicular aseptic impetigo that typically presents with sudden high fever and a rash characterized by a dense distribution of needle tip to soybean-sized pustules based on diffuse flushing, usually without mucosal and general organ damage (29). The incidence of AGEP is approximately 1–5/1,000,000/year. Approximately 90% of patients are on medication prior to disease onset, mainly in the form of antibiotics, with β-lactam medications being the most common (45). In a small number of patients, AGEP is caused by an infection or other factors (46) including other drugs that rheumatological patients may take to fight biologics-related infections, e.g., doxycycline or new anticoagulants (dabigatran) (47, 48). The specific pathogenesis of AGEP remains incompletely understood (49). HCQ has immunosuppressive, anti-inflammatory, and possibly antiviral properties, and common adverse effects include headache, various types of rashes, pruritus, and gastrointestinal dysfunctions, such as nausea, vomiting, and diarrhea (50). Several dermatological side-effects have been associated with the use of HCQ, including dryness, pigmentation, itching, alopecia, urticaria, measles, contact dermatitis, and worsening psoriasis, of which AGEP is one of the more serious but rare types (51). With the wide application of HCQ in clinical practice, HCQ-induced AGEP should be seriously considered.

The incubation time of AGEP is generally 3–30 d, and the median time of occurrence is 15 d after starting drug treatment. The time from drug exposure to the appearance of a rash depends on the causative drug; primitive mycin and amoxicillin can cause AGEP rash onset 1 d after the first dose, whereas other drugs trigger a reaction only after 1–2 weeks (52). For instance, carbamazepine-induced AGEP may take 5–35 d to manifest symptoms (53); when diltiazem induces AGEP, symptom onset occurs within 1–3 weeks (54). Previous cases of HCQ-induced AGEP ranged from 2 to 30 d between the initiation of HCQ treatment and rash onset (15, 52). Rash onset in the cases we reported ranged from 7 to 25 d, which is similar to results reported in the literature. In addition, studies have found that blood drug levels peak after 5–6 weeks of HCQ trial treatment, which may be the reason for the slow appearance of AGEP symptoms (55). This indicates that HCQ-related AGEP may be associated with its metabolic properties.

In the cases we studied, there were no mutations in the *IL-36RN* gene; however, we identified novel mutations in the *CARD14* gene in three cases at seven mutation sites. Of the three patients, one had SLE, one had RA, and one had RA with Sjogren’s syndrome. None of the three patients was in an active disease state, and there were no obvious symptoms of joint pain, swelling, skin allergies, or mouth ulcers. To date, *CARD14* gene mutations have been associated with various diseases that produce widespread pustular rashes, such as generalized pustular psoriasis (GPP), psoriatic arthritis, and pityriasis rosea (10, 56). In addition, mutations in other *CARD* genes are known to cause pustular skin disorders, similar to Burau syndrome (*CARD15*/*NOD2* mutations) (57). Owing to the limited number of reported cases, further research is needed to determine whether *CARD14* mutations form the molecular basis of AGEP.

The typical manifestations of AGEP include tens to hundreds of non-follicular pinhead-sized sterile pustules that appear rapidly as edematous erythema, generally originating on the face or intertriginous areas of the body, and rapidly spreading to the trunk and limbs. Histopathology on sub-corneal pustules located in the superficial layer of the epidermis reveals the presence of immune cells—mainly neutrophils and occasionally eosinophils—and an almost unchanged epidermis below the pustules, showing only infiltrated neutrophils and mild intercellular edema (2). In the five patients we reported, the rash initially appeared on the cheeks and facial area, joints of the extremities, scalp, and hands. Afterward, the rash spread rapidly to other parts of the body. All cases had facial involvement, which is consistent with the clinical presentation of AGEP. HCQ-related AGEP can be clearly distinguished from GPP—which recurs often—and there may be typical psoriasis lesions before the appearance of pustules; these appear on the original plaque or rash and can expand and fuse to form “pus lakes.” In addition, some patients with GPP have a family history, and the histopathological manifestations are psoriasis-like hyperplasia of the epidermis with keratosis, Kogoj micro-abscesses in the upper part of the spinous layer, and superficial dermal telangiectasia and tortuous vessels (58). In this sample, there was no family history of psoriasis, the rash appeared after HCQ treatment, and pustule formation was observed on histopathology, which is not the case in patients with GPP. Therefore, patients with suspected HCQ-induced AGEP may benefit from histopathology and skin biopsy.

AGEP is self-limiting and has a short disease course, and lesions generally disappear within 15 d of stopping the causative drug. However, HCQ-induced AGEP is prolonged and recurrent, ranging from 7–81 d (18, 37). As HCQ has a longer half-life, HCQ-associated AGEP lesions may last for longer, i.e., 1–2 months (19). All five patients in our sample were discharged within 15–45 d, similar to the results reported in the literature. The first principles and priorities of AGEP treatment are cessation of exposure to suspected triggers and symptomatic supportive care, avoiding antibiotics as much as possible, except in cases where co-infection is suspected; in such cases, antibiotics should be cautiously used (59). The use of topical emollients may aid the treatment of mild symptoms, whereas patients with severe symptoms require systemic steroids to relieve itching, inhibit telangiectasia and inflammation, and shorten the course of the disease; for recalcitrant disease, systemic therapy is recommended with dapsone and cyclosporine (60). Our patients received topical or systemic steroids and antihistamines, and the most severely ill patient also received mycophenolate and thalidomide, which rarely present with serious complications and sequelae. The mortality rate of AGEP is less than 5% and is often due to multi-organ dysfunction. Patients at high mortality risk typically have comorbidities or extensive skin lesions with mucosal involvement (4). The condition rarely recurs after treatment, but exposure to the same trigger may lead to recurrence. None of the patients we reported on had any recurrence after a 1–2 year-follow-up period.

Herein, we reported five cases of HCQ-induced AGEP. The need to distinguish HCQ-induced AGEP from GPP is of clinical concern. Compared to AGEP induced by other drugs, the latency and regression time of AGEP caused by HCQ are longer. AGEP often resolves with topical therapy and with drug discontinuation, although systemic steroid therapy may be necessary in some cases. In addition, a new *CARD14* mutation was identified in our study and was validated using Sanger sequencing.

## Data availability statement

The original contributions presented in the study are included in the article/supplementary material, further inquiries can be directed to the corresponding author.

## Ethics statement

The studies involving human participants were reviewed and approved by Medical Ethics Committee of the Second Affiliated Hospital of Guizhou University of Chinese Medicine. The patients/participants provided their written informed consent to participate in this study.

## Author contributions

W-kM and X-mYa: conceptualization. C-mC: methodology. Y-zY: software. W-kM, X-mYa, and FL: validation. HX: formal analysis. X-mYu: investigation. FL: data curation. FL: writing—first draft preparation. W-kM and X-mYa: writing—review and editing. All authors contributed to the article and approved the submitted version.

## Funding

The study was funded by the Guizhou Province Science and Technology Plan Project, Grant/Award Number: Oiankehe Platform Talent [2020] 2202, [2016] 5650; Guizhou Province Science and Technology Plan Project, Grant/Award Number: Guizhou Qiankehe Support [2021] General 006.

## Conflict of interest

The authors declare that the research was conducted in the absence of any commercial or financial relationships that could be construed as a potential conflict of interest.

## Publisher’s note

All claims expressed in this article are solely those of the authors and do not necessarily represent those of their affiliated organizations, or those of the publisher, the editors and the reviewers. Any product that may be evaluated in this article, or claim that may be made by its manufacturer, is not guaranteed or endorsed by the publisher.

## Footnotes

^1^ http://www.bgi-write.com
